# Mental Ill-Health and the Epidemiology of Representations

**DOI:** 10.3389/fpsyt.2018.00289

**Published:** 2018-07-19

**Authors:** Ladislav Kesner

**Affiliations:** National Institute of Mental Health, Klecany, Czechia

**Keywords:** mental ill-health, social psychiatry, epidemiology of representations, images, narratives

## Abstract

One of major challenges facing contemporary psychiatry is the insufficient grasp of relationship between individual and collective mental pathologies. A long tradition of diagnosing “mental illness” of society—exemplified by Erich Fromm—stands apart from approach of contemporary social psychiatry and is not perceived as relevant for psychiatric discourse. In this Perspective article, I argue that it is possible to uphold the idea of a supra-individual dimension to mental health, while avoiding the obvious pitfalls involved in categorical diagnosing of society as suffering from mental illness. I argue for an extended notion of public mental ill-health, which goes beyond the quantitative understanding of mental health as an aggregate of individual diseased minds captured in statistics, and which can be conceived as a dynamic, emergent property resulting from interactions of individual brains/minds in social space. Such a notion, in turn, presents a challenge of how to account for the interfacing between individual minds/brains and the collective mental phenomena. A suitable theoretical framework is provided by the notion of epidemiology of representations, originally formulated by cognitive anthropologist Dan Sperber. Within this framework, it is possible to highlight the role of public (material) representations in inter-individual transfer of mental representations and mental states. It is a suitable conceptual platform to explain how the troubling experiences with causal or mediating role on mental health, to a significant degree arise through a person's direct interaction with material representations and participation in collective mental states, again generated by material representations.

## Introduction

In a provocative study devoted to the “pathology of contemporary western society,” psychoanalyst, sociologist, and humanist philosopher Erich Fromm presented a sustained argument about why Western society should seriously question its collective sanity (1). Combining psychological erudition with philosophical acumen and the insights of an astute social critic, Fromm paints a bleak picture of the modern human predicament. From today's perspective, the aim of his *The Sane Society*—to diagnose the social mental illness of his time—seems both anachronistic and very timely. It looks anachronistic because, as I shall discuss below, it is removed from the methods and language of contemporary psychiatry. It is nevertheless timely in that most of Fromm's observations about the “pathologies of normalcy” and the “social character of contemporary man” sound all too familiar now, six decades after his book was first published. They strike a chord in our time as the media and public discourse have become increasingly saturated with views like Fromm's, with many thinkers trying to comprehend the great waves of irrationality and negative emotions that are flooding the public space and social media. Fromm's thoughts hit home at a time when it is plainly obvious that people's mental states are being massively manipulated and negative emotions intentionally elicited by media and by groups with vested political and economic interests in achieving particular and in some cases clearly sinister goals. They moreover resonate in a time when psychiatry itself is reassessing its theoretical paradigms, not least of all owing to a sobering realization about the limits of its biological bias (2, 3).

But does diagnosing modern-day society's mental pathologies in the spirit of Fromm have any heuristic value for contemporary psychiatry? I suggest that it might. Recent psychiatry has been taken to task for the decline of the “sociological imagination”—“a decline of theory and interest in the major questions that initially drove early sociological studies of mental disorder” (4). If this charge is taken seriously, then one challenge would be to construct conceptual frameworks for linking individual and collective dimensions of mental health.

Here, Fromm's perspective might provide a useful starting point.

## What is wrong with notions of collective mental ILL-health

Since the publication of his book, Fromm's effort to link social, and mental pathologies at the collective level has found some following in academic writing (5, 6). Within psychiatric discourse, it occasionally echoes in those works that view the current socio-political order of western societies as inherently detrimental to human well-being and psychological health (7, 8) and explicitly focus on “diseases of the collective *esprit de corps* of contemporary civilization” (9). Some social theorists and opinion makers have further extended this line of thought by linking social pathologies to what they perceive to be the pervasive psychological and mental malaise of contemporary society or even “a crisis of Western civilization” (10–12). Such “diagnoses” of collective mental ill-health stand apart from the rigorous science of contemporary psychiatry. A critic would object that they suffer from at least three related problems, each sufficient to render them invalid in the eyes of academic and clinical psychiatrists. First, diagnoses of collective mental ill-health are not reached impartially but in most cases emerge with just a thinly disguised ideological agenda. Almost invariably this involves criticism of capitalism, with global neoliberal capitalism being the usual culprit behind society's illness. Simple causality and correlation between the perception of social and mental pathologies are sought and established, while more nuanced considerations are swept under the carpet. Tellingly, Mark Fisher accuses neo-liberalism of generating a “mental health plague” (10) while political theorist Franco Berardi writes of the “suicidal form of the neoliberal will to win” that is permeating contemporary culture, together with the phenomenology of panic, aggression, and resultant violence, the subjects of his book being heroes “of an age of nihilism and spectacular stupidity: the age of financial capitalism” (11).

Secondly, these views suffer from a negative bias. Diagnoses of collective mental malaises are often based on their authors' interpretation of media representations, which tend to produce a negatively biased picture of the state of the world. Events, possibly caused by an individual mental disorder, all too easily acquire the symptomatic value. Thus, tragic incidents, such as the suicide of the German pilot who crashed his plane, killing all passengers on board (or other mass murders discussed by Berardi), are quickly established as both a symptom and a consequence of the collective psychopathology of an “increasingly anxious and depressed society”. Both these problems, and in particular the shortcut from socio-economic order to mental disorder, have plagued even the most serious meditations on collective mental health, such as Karl Jaspers's observations on modern anxiety (13) or Fromm's own book.

Thirdly, and most importantly, notions about collective mental states, which were once popular but are nowadays rather marginalized, even in social sciences, have never gained much traction in psychiatry, and even less so within the realm of biological psychiatry, which is keen on rebranding mental disorders as “brain disorders”(14, 15). After all, there are no collective brains.

## Two complementary notions of collective mental (ILL) health

Diagnoses of collective mental ill-health, in the tradition of Fromm, thus seem inherently at odds, and impossible to reconcile with the nuanced and sophisticated methods of social psychiatry and psychiatric epidemiology, which provide increasingly accurate assessments of the prevalence of mental disorders and insights into the converging effects of genetic and socio-environmental and socio-economic risk factors and stressors. Public or “global mental health” epidemiology (16, 17) relies on objectifiable measures of mental disease obtained from large-scale mental health surveys and epidemiological studies (18, 19) and, more recently, the use of epidemiological “big data” (20, 21). But it has also been argued that “…much of the recent social epidemiology of mental disorder has been largely atheoretical, seeking simply to document the rates of occurrence of specific mental disorders and their socio-demographic correlates” [(4), p. 57]. The prevailing conceptualization of mental health, which is based on a quantitative approach and nosological categories of psychiatry and thus depending on statistically validated cases of reported mental disorders, is, for all its virtues, clearly limited by the nature of its data. What it cannot capture is the broader, collective dimension, implicit in Fromm's diagnoses. Rethinking the Frommian perspective is thus clearly germane to current debates and contentions about the reliability and validity of measures of mental health and illness (16), as well as to ongoing discussions about the role of the social dimension in the etiology of psychiatric disorders and the view that social factors are not well acknowledged by the dominant model of biological psychiatry (22–24). But it should be possible to uphold the idea of a supra-individual dimension to mental health, while avoiding the pitfalls involved in diagnosing—in Fromm's manner—an entire society as suffering from mental illness. In concrete terms this would involve considering a notion of disordered collective mental state (25) as an entity that at any given moment is composed of all the statistically reported cases of mental illness, as well as (i) instances of (as yet) undiagnosed and untreated cases of “harmful mental dysfunctions” (26, 27) and (ii) individual cases of dysfunctional psychic life, which lie in the gray zone between normalcy and pathology or fluctuate between the two. The latter cases have not (yet) achieved the status of a diagnosis and do not fulfill the nosological criteria of mental disease; therefore, they remaining below the radar of psychiatric epidemiology, but nonetheless manifest in maladaptive, individually and socially harmful behaviors that affect subjects' social interactions and can be readily observed as a sign and symptom of overarching malaise. Such a notion is in line with the view that the boundaries of mental disease are fluctuating (28) and in particular is consistent with the well-established dual-factor or two-continua models of mental health and illness (29–31) and dimensional model of psychopathology (32).

It is a matter of consensus that human minds and brains are not stand-alone fixtures, but are modified in and through interactions with other brains. If this is so, one should make room for the coexistence of two related, yet ontologically distinct, notions of public mental ill-health: (i) the one that is currently circulating in social psychiatry and epidemiology, based on hard epidemiological and statistical data, and (ii) a second notion, which goes beyond and above the quantitative understanding of mental health as an aggregate of individual (diseased) minds/brains captured in statistics, and which can be conceived *as a dynamic, fluctuating emergent property resulting from interactions of individual brains/minds in social space*. This type of inclusive, dynamic notion of mental ill-health creates an immediate theoretical challenge: the central question for the philosophy and theory of psychiatry is not just *how mental states (normal or disordered) are related to brain states* (2, 33, 34). Rather, what is at stake is how we theorize the relationship between *individual minds/brains and the collective mental phenomena* or *individual and collective mental health*. A potentially rewarding framework is provided by the epidemiology of representations, a notion introduced by cognitive anthropologist Dan Sperber to describe the realization and implementation of mental content in the material world (35–37).

## The epidemiology of representations

In its most basic terms, the epidemiology of representations can be formulated as follows: individual humans have their own subjective mental representations, which are underlined by specific patterns of neural representations—that is to say, by spatiotemporal patterns of neural activity demarcated from the background activity of the brain. Some interactions among individuals in the social space result in the creation and dissemination of collective representations in multiple minds/brains, which recursively shape the structure and content of individual consciousness and probably the functional microstructure of individual brains as well (38, 39). Collective mental representations arise and propagate along two main trajectories. First, through direct “on-line” interactions involving various kinds of exchanges, synchronizations, attunements, and emotional contagions among people. Recent research has provided fascinating new evidence on the interindividual transfer of mental states, such as mass-scale emotional contagions that proceed through social networks (40) or the spread of happiness (41) or depressive symptoms and moods (42) along social networks. Other studies have demonstrated that certain mental states may be underlined by the synchronization of brain activity within a given collective (43). On a broader scale, there are cycles of mutual constitution of individual brains/minds and culture that involve a “looping effect,” in which culture shapes the brain by contextualizing behavior, and the brain fits and modifies culture via behavioral influences (44–46).

However, the inter-individual transfer of mental representations and mental states also crucially propagates along a second trajectory, namely through public (material) representations. Some individual mental representations (beliefs, ideas, attitudes, stereotypes, presumptions, memories, etc.) are transformed or “offloaded” into materially instantiated public representations (images, symbols, and texts), which, spreading through social and virtual space, in turn activate, disseminate, and stabilize mental representations among a certain (and often large) number of people. Sperber argued that the ontology of this transfer resembles epidemiology. The epidemiology metaphor appears particularly apt for our purpose, as it captures both the potential of public representations to quickly affect a large population and their viral effect on individual mental states. At the same time, it is compatible with current sociological models of collective subjectivities (47) and models of social cognition or macrocognition (48, 49). Extending Sperber's model appears eminently useful for psychiatry.

As many authors have forcefully argued, the genetic and neurobiological factors of mental disorders are profoundly, if not decisively, shaped by the psychosocial environment and life experiences (4, 50, 51). Indeed, the blind spot of strictly biomedical approaches and neuroimaging of mental disorder may be the failure to consider life experience (51, 52). As Paradiso and Rudrauf recently argued: “.the adequate level of integration is precisely that of a subject with genetic vulnerability and with a history and place assigned or imagined to be assigned by others, living in a world of representations while building a narrative about them, and coping with conflicts and dissonances at multiple levels” [53, p. 72].What has not been sufficiently recognized and theorized is the fact that a person's real and symbolic relationship with the world, and in particular the troubling and traumatic experiences with causal or mediating role on mental health, to a significant degree arise through a person's direct interaction with material representations.

## Pathways and mechanisms

There are a number of challenges involved in specifying the pathways and mechanisms by which the epidemiology of representations impacts mental health. Given the limited space here, I shall briefly focus on the main ones. First, material representations span several hierarchical levels. Humans are affected on a cognitive and neural level by (i) individual images, symbols, or texts. Most of these, however, are embedded and thus perceived within (ii) more complex image-texts and symbolic structures. These, in turn, are often incorporated into and participate in (iii) complex narratives and metanarratives. Marshal McLuhan's famous dictum that the “medium is the message” is pertinent here, for it is not just *content*, but also the *structural features* of the various media platforms that deliver public representations that can exercise a negative impact on mental health. There is, e.g., some evidence that addiction to social media and the Internet is associated with psychiatric comorbidity (54–56). Neuronal and cognitive mechanisms of (mostly autonomic, reactive) response to individual pictorial or verbal stimuli have been extensively studied and specific abnormalities of such responses are routinely associated with specific psychiatric disorders, such as depression, PTSD, alexithymia, and others [e.g., (57–59)]. However, the focus on dysfunction of low-level perceptual and reflexive emotional processes in the psychiatric population may in fact have little bearing on mechanisms of psychiatric illness (53). There is also growing evidence of the role of the media in generating negative mental states—for example, the effects of “media amplification,” in which exposure to traumatic disaster news triggers anxiety symptoms, stress disorders, and psychopathology (60–63). In these cases, representations affect their consumers directly.

Much less is known about responses to material representations on the conscious and behavioral levels and particularly about the dynamics of tripartite interaction among individual and collective mental and material representations. What needs to be unraveled are the mechanisms involved in the cyclical effect, whereby individual cognitive and affective mental states determine people's response to and interpretation of public representations, including massive amount of weaponized political messages and targeted disinformations. Consumption of a broad spectrum of these representations, spanning both unconscious/reflexive and conscious/reflective response, then elicits affective, and cognitive responses, which directly impact emotion, and mood states and through them recursively shape the formation and circulation of attitudes, stereotypes, prejudices, ideas, and other mental contents.

Different trajectories are at work: in one, the dissemination of certain public representations generates collective mental representations, with the potential to impact affective mental states in large groups. Thus, e.g., media-inflicted dehumanized stereotypes of an enemy ethnicity have been proven to be potential triggers of collective genocidal rage, manifested in atrocities, such as those observed in Rwanda and the former Yugoslavia. More importantly for out topic here, the effects of media are mostly less extreme and cataclysmic, but all the more pervasive. Public representations are (often in subtle ways) capable of generating and exacerbating negative emotions, anxieties, frustrations, and anhedonia, some of which are transferred into collective mental states and behavioral outcomes. Indeed, influential current models, which single out increasing uncertainty and negative anticipation as a causal factor in the pathogenesis of anxiety (64, 65) and depression (66) should be productively extended by recognizing the critical role that material representations play in these mechanisms, specifically by triggering, and augmenting uncertainty, negative anticipation, and feelings of anhedonia and helplessness.

Furthermore, it is necessary to consider how the epidemiology of representations relates to adverse social risk factors and social stressors, whose impact on mental health has been extensively documented (67–69). Social disorganization, rapid social change, socio-economic disadvantage, and deprivation, increased competition and inequality, and social isolation and loneliness are traumatic experiences that act as facilitators and triggers of mental illness. Remembering that people exist in both a real and a symbolic relationship with the world, it is imperative to fully acknowledge that most of these factors are also mediated and exacerbated by mutually interacting collective mental and material representations. Research needs to focus on analyzing how (epidemiologically-operating) representations disseminate and amplify other social stressors and adversities.

## Conclusion

One of the major challenges facing psychiatry today is to analyse how specific social contexts and experiences combine with biological and genetic mechanisms in the etiology of mental disorders. To do this, as I have tried to argue here, research needs to account for the dynamics of the tripartite interaction between individual and collective mental representations and public (material) representations. Admittedly, such a perspective may be inherently difficult for the prevailing model of psychiatry today, embedded as it is in an individual-level perspective of brain disease (70). Finally, it bears emphasizing that material representations, while having powerful effects on humans, impacting them across a range of levels, from the neural to the social, have no agency of their own. Rather, they are intentionally created, and disseminated by human actors, often to elicit specific patterns of behavior through their impact on mental states. The task of accounting for the impact of public representations on mental health thus extends to seeking to understand the motives and intentions of these actors. Such a task, admittedly, is beyond the purview of psychiatry alone. But if dialogue between the neurosciences and other (anthropological, social) sciences is required in order to explore the etiopathogenesis of patterns of mental disorders and to elucidate higher-order psychological and cultural factors in mental disorders (71), then the epidemiology of representations constitutes a prime example of a framework within which this kind of dialogue can occur.

## Author contributions

The author confirms being the sole contributor of this work and approved it for publication.

### Conflict of interest statement

The author declares that the research was conducted in the absence of any commercial or financial relationships that could be construed as a potential conflict of interest.
